# OEA loaded liposomes with the neuroprotective effect for stroke therapy

**DOI:** 10.3389/fchem.2022.1014208

**Published:** 2022-09-08

**Authors:** Shichao Wu, Xiangrui Yang

**Affiliations:** ^1^ Department of Nuclear Medicine, Xiangya Hosptal, Central South University, Changsha, Hunan, China; ^2^ Key Laboratory of Nanobiological Technology of National Health Commission, Xiangya Hospital, Central South University, Changsha, Hunan, China; ^3^ National Clinical Research Center for Geriatric Disorders, Xiangya Hospital, Central South University, Changsha, Hunan, China

**Keywords:** OEA, stroke, neurprotective effect, ischemia reperfusion, drug delivery

## Abstract

With high mortality, stroke has become a serious threat to human health. Nevertheless, the strategy for stroke therapy is quite limited in the clinic till now. In this research, we prepared a novel neuroprotective nanoformulation (OEA Liposomes) via encapsulating endogenous N-oleoylethanolamine (OEA) in liposomes for intravenous administration. The formulation largely increased the solubility and bioavailability of OEA. Then the following systematic experiments stated the excellent neuroprotective effect of OEA Liposomes *in vivo*. The survival rate of the nanodrug group was largely increased to 75%, while that of the Middle Cerebral Artery Occlusion (MCAO) group was only 41.7%. And the severe neurological functional deficit of the MCAO rats was also significantly improved. What’s more, the OEA Liposomes could inhibit the apoptosis of neurons and the inflammation of reperfusion to a very slight level, indicating their outstanding neuroprotective effect. These results indicated that the OEA Liposomes have a great potential for clinic anti-stroke application.

## Introduction

With high mortality, stroke has been a leading cause of death worldwide. Plus the high rate of long-term disability, stroke has become a serious threat to human health (Centers for Disease Control and Prevention, 2015) (Centers for Disease Control and Prevention (CDC), 2009). What’s worse, the research in clinical stroke trials progressed slowly or stalled, and only a few effective formulations was available in the clinic now (Lo et al., 2003; Winstein et al., 2016).

When the research of neuroprotective drugs still remains elusive, our research group found that *N*-oleoylethanolane possessed an excellent neuroprotective effect (Zhou et al., 2012; Yang et al., 2016; Zhou et al., 2017a). As an endogenous peroxisome proliferator-activated receptor alpha (PPAR-α) agonist, OEA could protect against acute and chronic ischemic injury (Yang et al., 2019). The mechanism is that the activation of PPAR *α* could significantly inhibit the inflammation induced by ischemia reperfusion, which is one of the primary causes of severe brain damage (Zhou et al., 2017b; Luo et al., 2019). More strikingly, chronic administration of OEA improved spatial cognitive impairments and even promoted neurogenesis and neuroplasticity in MCAO rats (Yang et al., 2015a). Apart from other synthetic neuroprotective drugs, OEA is an endogenous compound and possesses superior effectiveness and safety. Therefore, OEA is a promising neuro-protector and may have potential applications in stroke therapy.

However, the solubility of OEA is extremely low, which has been a major obstacle to its absorption. And OEA could not take part in the salt-forming reaction, which is a common strategy to enhance the solubility of the drug candidate in formulation development (Lipinski, 2000). These poorly water-soluble candidates are usually dismissed at the beginning of pharmacological characterization (Porter et al., 2008). However, it is precious to find an endogenous compound with a nice neuroprotective effect. Hence, nanoparticles drug delivery system (NDDS) was employed to improve the pharmaceutical properties of OEA (Chen et al., 2018; Wu et al., 2022). Among all kinds of NDDS, liposomes or lipidic nanoparticles are the first nanomedicine delivery system passed the clinical trials, which has been an established technology platform with considerable clinical acceptance (Wicki et al., 2015). More and more liposomal formulations are in the pipeline now, which would enter the market in the near future (Tran et al., 2017). Through decades of research, many important technical advances in liposome preparation were developed, such as remote drug loading, long-circulating liposomes, nucleic acid loaded liposomes, and so on (Lukyanov and Torchilin, 2004). One particularly important advance was the extrusion for homogeneous size, which played a paramount role in the transition of the liposomes from concept to clinical application (Allen and Cullis, 2013; Bulbake et al., 2017). What’s more, many complex liposomes have been designed and prepared, such as ligand-targeted liposomes (Lv et al., 2018), and triggered release liposomes (Zhang et al., 2018; Ansari et al., 2021). Although they were not applied in the clinic now, these advances led to numerous clinical trials and more potential applications in the delivery of anti-cancer (Cao et al., 2016; Kiaie et al., 2020), anti-biotic (Shen et al., 2022), anesthetics (Ruokonen et al., 2017), and anti-inflammatory drugs (Mokdad et al., 2022).

In this paper, OEA was successfully encapsulated in PEGylated liposomes to improve its pharmaceutical properties. Through extrusion, the OEA Liposomes possessed a homogeneous and uniform size of 97.6 ± 4.8 nm, which met the standard of injection and could be intravenously administrated to the rats. The *in vitro* drug release study indicated the controlled and sustained drug release property of OEA Liposomes. And the *in vivo* neuroprotective effects were systemically evaluated. The results suggested that the OEA Liposomes could greatly increase the survival rate of MCAO rats and improve their neurological functional deficit scores. Significantly, the OEA Liposomes could inhibit the apoptosis of neurons and the inflammation of reperfusion to a very slight level, indicating their outstanding neuroprotective effect. The research stated that the OEA Liposomes might process the clinic application of OEA and become a promising anti-stroke NDDS.

## Materials and methods

### Materials

OEA (purity grade > 90.0%), SPC (purity grade >90%), and cholesterin (purity grade >90%) were purchased from Shanghai Macklin Biochemical Co., Ltd. (Shanghai, China). DSPE-PEG (MWCO 2000) was obtained from Ponsure Biotechnology Co., Ltd. (Shanghai, China). Trichloromethane was purchased from Sinopharm Chemical Reagent Co., Ltd. All chemical reagents were of analytical grade and used without further purification unless otherwise stated.

### Preparation and characterization of OEA liposomes

The OEA Liposomes were prepared *via* the thin film hydration method coupled with sequential extrusion. Briefly, OEA (5 mg), cholesterin (5 mg), SPC (5 mg), and DSPE-PEG (35 mg) were dissolved in trichloromethane, which was evaporated under vacuum for 8 h to prepare a dry film. PBS (100 ml, pH 7.4) was added under sonication for 10 min, and the dispersion was intensely stirred for 2 h. Lastly, the dispersion was extruded through polycarbonate filters of decreasing pore diameters of 0.8 μm/0.4 μm/0.2 μm (three passages through each) to obtain liposomes with uniform size.

Morphology of the OEA Liposomes was examined by TEM (Tecnai G2 F20, United States). The Size of the OEA Liposomes was determined by Malvern Zetasizer Nano-ZS (Malvern Instruments, Malvern). The drug loading content of OEA in OEA Liposomes was determined by liquid chromatography-mass spectrometry (LCMS ORBITRAP VELOS PRO ETD, United States). The drug loading and encapsulation efficiency was calculated by Eqs 1, 2, respectively.

Drug loading content of OEA (%)=(weight of OEA in Liposomes)/(weight of Liposomes)×100% (1)

Entrapment efficiency of OEA (%)=(weight of OEA in Liposomes)/(weight of feeding OEA)×100% (2)

### The *in vitro* drug release study

The *in vitro* drug release studies of OEA Liposomes were performed via the dialysis technique. Free OEA and OEA Liposomes were dispersed in PBS (10.0 ml) and placed in dialysis bags (MWCO 3500 Da). Then, the bags were immersed in PBS (150 ml, pH 7.4) containing 0.5% w/v Tween 80 and continuously oscillated in a shaker incubator (180 rpm, 37°C). All samples were assayed by HPLC.

### Drug administration

The OEA Liposomes (0.5 mg/ml) were dissolved in saline under sonication. OEA (0.20 mg/ml) and Tween 80 were dissolved in saline under strong shaking. Drugs (1.5 mg/kg, iv) were administered with reperfusion, and daily for 14 consecutive days after reperfusion.

### Preparation of the focal cerebral ischemia model

Focal cerebral ischemia was induced by middle cerebral artery occlusion in adult male SD rats (200–220 g), as previously described. Briefly, rats were anesthetized with chloral hydrate (350 mg/kg, ip). The silicon rubber-coated nylon monofilament was inserted into the left internal carotid artery (ICA) through the external carotid stump (ECA), and passed the ECA/ICA bifurcation to occlude the origin of the middle cerebral artery (MCA) at the junction of the circle of Willis. The monofilament was kept for 90 min and then withdrawn. Sham-operated rats were treated with an identical surgery without inserting the intraluminal filament. Throughout the procedure, body temperature was maintained at 37°C ± 0.5°C. Animals were excluded if hemorrhage was found in the brain slices or at the base of the circle of Willis during postmortem examination.

### Measurement of the survival rate

The survival rate of the rats was recorded. (*n* = 12 rats for the sham group and the OEA Liposomes group, and *n* = 24 rats for the MCAO group and the free OEA group). The rats were involved in the following experiments.

### Evaluation of neurological deficit

At 1, 3, 5, 7, and 14 days after reperfusion, rats (*n* = 8) were evaluated neurologically with the Garcia method (Garcia et al., 1995; Shimamura et al., 2006; Sozen et al., 2009) by one single examiner who was blinded to the animal groups.

### Immunofluorescence staining and cell counting

The MCAO rats (21 days after reperfusion) were sacrificed and their brains were stained with Immunofluorescence (TUNEL, Iba-1 and GFAP). The number of TUNEL, GFAP, or Iba1 positive cells was obtained by fluorescence confocal microscopy (EX61, Olympus, Tokyo, Japan). The positive area was counted in three randomly chosen squares of identical size (460 μm × 460 μm).

### Statistical analysis

The statistical significance of treatment outcomes was assessed using one-way/two-way analysis of variance for the differences within treatments followed by Tukey’s *post hoc* test (Prism 7 for Windows, GraphPad Software Inc., United States); *p* < 0.05 was considered statistically significant in all analyses (95% confidence level).

## Results and discussions

### Preparation and characterization of OEA liposomes

**FIGURE 1:**
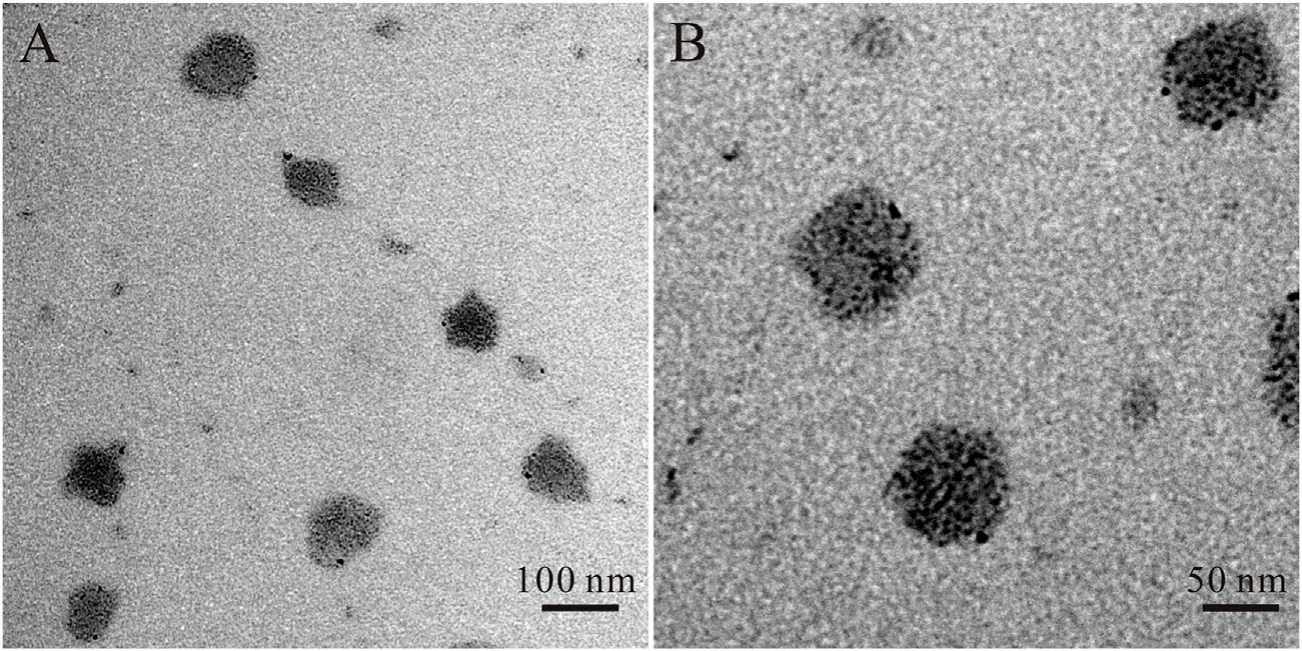
The TEM images of OEA Liposomes.

**FIGURE 2:**
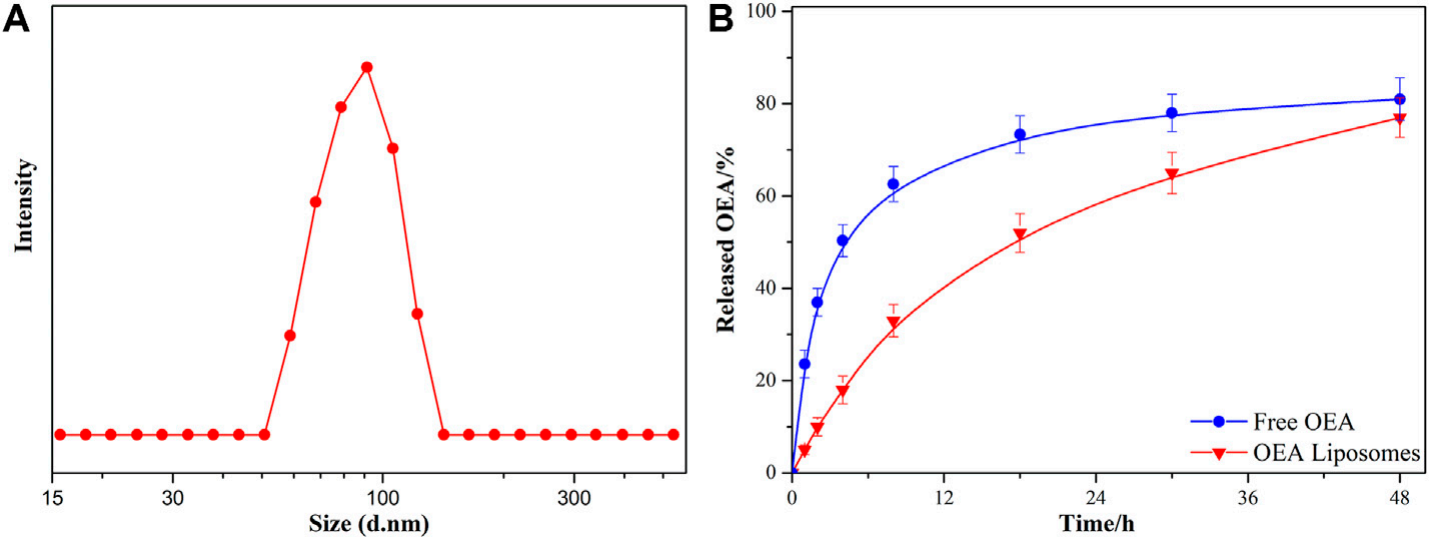
The size **(A)** and *in vitro* drug release profiles **(B)** of OEA Liposomes.

The OEA Liposomes were prepared via the thin film hydration method coupled with sequential extrusion. Simply, OEA (5 mg), cholesterin (5 mg), SPC (5 mg), and DSPE-PEG (35 mg) were dissolved in trichloromethane, which was evaporated under vacuum for 8 h to prepare a dry film. Then, the film was hydrated by adding buffer (pH 7.4) and intensely stirring. Last, the dispersion was extruded through polycarbonate filters of decreasing pore diameters of 0.8 μm/0.4 μm/0.2 μm (three passages through each) to obtain liposomes with uniform size. The TEM images indicated that the OEA Liposomes were nearly spherical in shape and possessed a size of approximately 70 nm (Figure 1). The DLS result indicated that the OEA Liposomes possessed a hydrodynamic size of 97.6 ± 4.8 nm and a PDI of 0.193 (Figure 2A). Since the retention of highly hydrophobic drugs in liposomes was always problematic (Zhigaltsev et al., 2010; Wu et al., 2021), the drug loading and loading efficiency of highly hydrophobic OEA was relatively low. The drug loading of OEA in the liposomes was 3.32% ± 0.46%. The loading efficiency of OEA in the liposomes were determined to be 28.3% ± 3. 4%.

### *In vitro* drug release of OEA liposomes

The *in vitro* release study of OEA from OEA Liposomes was performed *via* a dialysis technique, alongside with free OEA. All samples were assayed by HPLC. Since OEA was a highly hydrophobic compound, 0.5% w/v Tween 80 was added to create a sink condition (Wang et al., 2011). As shown in Figure 2B, about 50% of the OEA was released at 4 h, indicating the severe burst release of free OEA. And the whole release of free OEA exhibited an evident “fast followed by slow” type. In comparison, the OEA Liposomes showed a remarkably prolonged and sustained release profile during the release period. Since the drug release was largely affected by the drug loading, the low content of OEA in OEA Liposomes also contributed to the sustained drug release (Yang et al., 2015b). Plus the controlled release property of liposomes, the *in vitro* release of OEA Liposomes was close to a zero-order release.

### The survival rate

**FIGURE 3:**
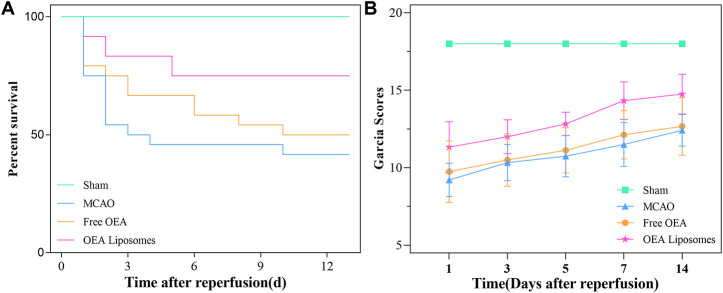
The survival rate **(A)** and the Garcia scores **(B)** of the MCAO rats administrated with the indicated formulations.

As an acute central nervous system disease, ischemic stroke possessed high mortality. Hence, reducing death rates must be the primary task to a formulation for stroke. So the survival rate of the MCAO rats injected with the indicated formulations {0.9% NaCl [MCAO], free OEA, and OEA Liposomes, [(OEA) = 1.5 mg/kg]} was recorded. As shown in Figure 3A, only 41.7% of the MCAO rats without treatment could survive for 14 days, indicating the severe damage and high mortality of MCAO. The injection of free OEA could not significantly improve this data, and there were still more than half of the MCAO rats who died within 14 days. In comparison, OEA Liposomes could obviously increase the survival rate to 75%. Only three MCAO rats injected with OEA Liposomes died from the cerebral injury. The result indicated that the administration of OEA Liposomes could build up resistance to cerebral ischemia significantly and extend the time window for beneficial reperfusion (Campbell et al., 2019).

### The behavioral assessment

In the clinic, the behavior of the patients was one of the clear indicators for illness assessment (Shea et al., 1992). Then the neurological functional deficit scores of the MCAO rats were assessed *via* the Garcia method. The Garcia scores of the MCAO rats administrated with the indicated formulations were evaluated at 1, 3, 5, 7, and 14 days after reperfusion. As shown in Figure 3B, the sham-operated rats got full marks throughout the estimate, indicating their normal brain condition. The MCAO rats without treatment got about nine points at 1 day after reperfusion, stating their deficient neurological function. Although the Garcia score of the rats in this group increased gradually, they did not get more than 13 points in the next assessments. The result suggested that the brain damage caused by MCAO was severe and hard to recover. And the MCAO rats injected with free OEA got almost the same scores as those without treatment, indicating that the administration of OEA did not obviously improve the neurological functional deficit caused by MCAO. On the contrary, the MCAO rats administrated with OEA Liposomes got significantly higher scores than the other operation rats (*p* < 0.01). The results illustrated that OEA could significantly mitigate and ameliorate the neurological function deficit caused by MCAO and the subsequent reperfusion.

### The apoptosis of neurons

**FIGURE 4:**
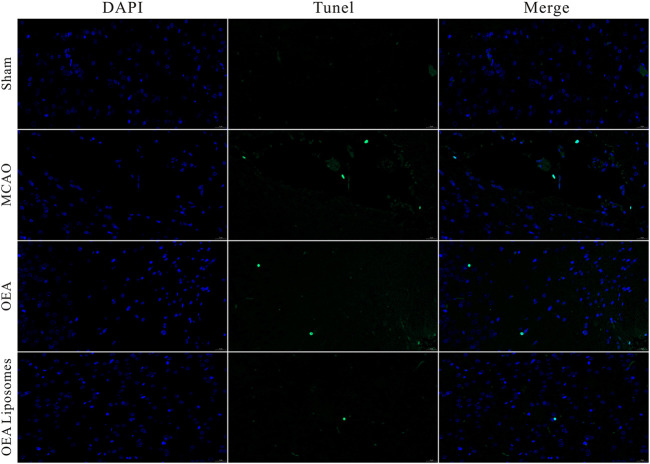
Representative pictures of the cortex stained with TUNEL.

**FIGURE 5:**
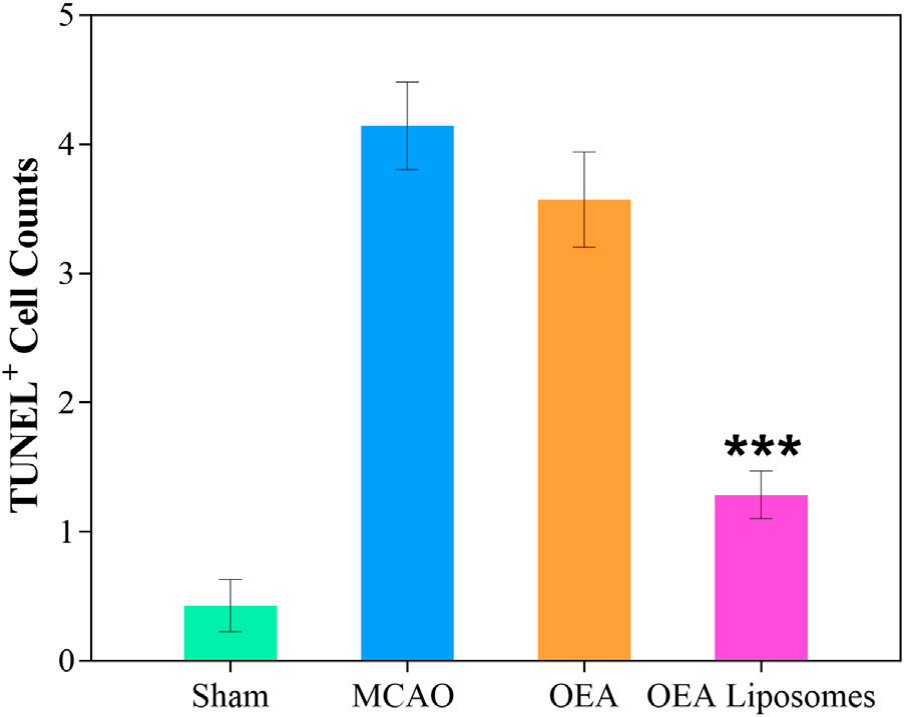
The statistical data (D) of the cortex stained with TUNEL. Data are expressed as mean ± SEM. *** = *p* < 0.001, compared to MCAO group).

The neurological function deficit might come from the apoptosis of neurons, which were really hard to recover and would lead to intractable sequelae (Dirnagl et al., 1999; Tuo et al., 2022). Herein, TUNEL staining was employed to evaluate the ischemia reperfusion induced apoptosis in the cerebral cortex. As shown in Figures 4, 5, there was no apoptotic cell in the cerebral cortex of the sham-operated rats, suggesting their good brain condition. And significantly increased apoptotic cells were observed in the rats’ brains of the MCAO group and OEA group. The result indicated that the ischemia reperfusion indeed induced the apoptosis of the neurons, and this could not be improved by the injection of free OEA. On the contrary, the administration of OEA Liposomes could obviously protect the neurons from apoptosis, and only one apoptotic cell was observed in the field. The result indicated the excellent neuroprotective effect of OEA Liposomes.

### The inflammation

**FIGURE 6:**
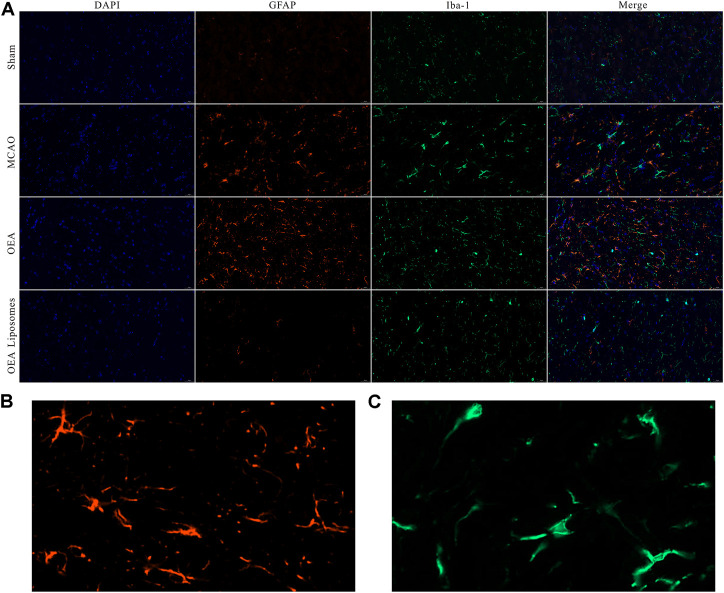
**(A)** The area of IBA-1 and GFAP positive stain from the cortex around the ischemic focus. **(B–C)** The enlarged pictures of IBA-1 and GFAP positive area.

**FIGURE 7:**
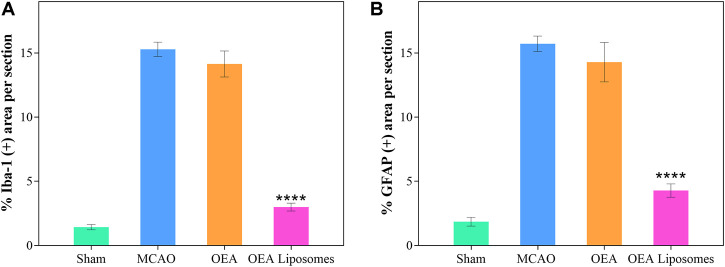
The statistical data of IBA-1 **(A)** and GFAP **(B)** positive stain from the cortex around the ischemic focus. Data are expressed as mean ± SEM. (**** = *p* < 0.0001, compared to MCAO group).

According to our previous study, the OEA works as a neuroprotective candidate by inhibiting the inflammation, which is one of the primary causes of severe brain damage. The inflammation in the brain was mainly mediated by microglia and astrocyte, which would be specifically marked by Iba-1 and GFAP, respectively. Here, the Iba-1 and GFAP in the cortex were marked to access the inflammation after reperfusion. As depicted in Figures 6, 7, both markers indicated that the brain from the rats of the MCAO group or the OEA group possessed a largely increased inflammation compared with the Sham group. However, the administration of OEA Liposomes could significantly inhibit the inflammation caused by ischemia reperfusion, which was almost as slight as that of the Sham group. The result suggested that the OEA Liposomes could inhibit the inflammation of ischemia reperfusion to a very slight level, and therefore, provide significant neuroprotective effects.

## Conclusion

In summary, the study demonstrates OEA loaded liposomes for efficient stroke therapy. The highly hydrophobic property of OEA largely limited the clinic application of OEA. And the encapsulation of OEA into liposomes obviously increased its solubility and provide a sustained drug release property. Hence, the bioavailability was largely enhanced. The *in vivo* assessments indicated that the survival rate and the behavioral score of the MCAO rats were significantly improved *via* the administration of OEA Liposomes. What’s more, the apoptosis of the neurons and the inflammation in the cerebral cortex were significantly inhibited to a slight level. These results strongly suggested that the OEA Liposomes had potential applications for stroke therapy, and might give impetus to the clinic use of OEA.

## Data Availability

The raw data supporting the conclusion of this article will be made available by the authors, without undue reservation.
